# Lung Cancer in Women—Sociodemographic, Clinical and Psychological Characteristics with Comparison to Men

**DOI:** 10.3390/jcm13051450

**Published:** 2024-03-02

**Authors:** Anna Trojnar, Magdalena Knetki-Wróblewska, Piotr Sobieraj, Joanna Domagała-Kulawik

**Affiliations:** 1 Department of Internal Medicine, Pulmonary Diseases and Allergy, Medical University of Warsaw, 02-091 Warsaw, Poland; magdalena.knetki-wroblewska@nio.gov.pl; 2 Department of Lung Cancer and Chest Tumors, Maria Skłodowska-Curie National Research Institute of Oncology, 02-781 Warsaw, Poland; 3 Department of Internal Medicine, Hypertension and Vascular Diseases, Medical University of Warsaw, 02-091 Warsaw, Poland; 4 Maria Skłodowska-Curie Medical Academy, Institute of Clinical Sciences, 00-136 Warsaw, Poland; joanna.domagala-kulawik@uczelniamedyczna.com.pl

**Keywords:** lung cancer, sex differences, psychological evaluation, females, smoking, psychological questionnaire

## Abstract

(1) **Background:** There is a difference in the course of lung cancer between women and men. Therefore, there is a need to evaluate various factors in the patient population treated in daily practice. The purpose of this study was to analyze the clinical, sociodemographic and psychological aspects of female lung cancer. To better express the results, we compared women and men. (2) **Methods**: Consecutive patients with a history of lung cancer treatment admitted to the outpatient oncology clinic (Department of Lung Cancer and Chest Tumours, Maria Skłodowska-Curie National Research Institute of Oncology in Warsaw) and the Department of Internal Medicine, Pulmonary Diseases and Allergy, were enrolled. We conducted analyses of the clinical, psychological and socioeconomic factors of women with lung cancer treated in everyday practice, including a comparison with a group of men. Demographic data were collected from a self-administered questionnaire. We used the Perceived Stress Scale (PSS-10) and Acceptance of Illness Scale (AIS) questionnaires for psychological evaluation. (3) **Results**: A total of 100 patients with confirmed primary lung cancer with a history of treatment were enrolled in the study (50 women and 50 men). We found a significantly shorter history of smoking in the group of women; at the same time, there were no differences in the reported incidence of COPD. Despite comparable results to men on the psychological questionnaire (PSS-10, AIS), women more often reported a willingness to be supported by a psychologist or psychiatrist due to lung cancer. However, they did not decide to consult them more often than men. Immunotherapy was a significantly less frequently used method in women. (4) **Conclusions**: We should be more active in finding out the willingness to consult a psychologist or psychiatrist among women with lung cancer. The diagnosis of COPD should be considered more often among women due to the lack of differences in the reported incidence of COPD between men and women, despite a clear contrast in the number of pack-years.

## 1. Background

Lung cancer is the leading reason for mortality from oncological causes. It remains a growing health problem, with smoking as the main risk factor. Current data present an incidence (age-standarized rate per 100,000 people) among men of 31.5, and women 14.6 and mortality among men of 25.9, and women 11.2 [1]. More than 84% of women and 90% of men with a new diagnosis of lung cancer have ever smoked [2]. Fortunately, in the last 20 years, current tobacco users decreased by approximately 10% worldwide, and this rate is projected to decline further [3]. The global reduction in smoking prevalence, the availability of screening programs and the use of targeted molecular therapies and immune checkpoints inhibitors have improved incidence and mortality outcomes over the past two decades in developed countries [4,5].

Women with lung cancer have certain characteristics that are different from men [6]. Females tend to have later adopted and then ceased tobacco smoking compared to males [7]. Lung cancer in women who have never smoked is diagnosed more often compared to men in all age groups, race/ethnicities and most histological types [2,8]. Adenocarcinoma is the histological type most frequently diagnosed in this group [9]. Women are more often predisposed to lung cancer mutations (EGFR) and rearrangements (ALK, BRAF) [8,10]. Another factor includes hormonal influence—an overexpression of estrogen receptors in adenocarcinomas, the role of aromatase in carcinogenesis of lung cancer and a relationship between estrogens and EGFR mutations [11].

Socioeconomic status remains an important aspect among patients with lung cancer. Based on systematic reviews by Redondo-Sachez, the risk of lung cancer is 50–80% higher among people with a lower socioeconomic status (smoking is responsible for 40–70% of this risk). A lower socioeconomic status in childhood can have a greater impact on cancer mortality in adulthood (at least due to smoking exposure) [12]. Among the articles included in the above systemic review, only one study was stratified by sex and reported similar results in both sexes [13].

Patients with lung cancer have the highest incidence of mood and anxiety disorders compared to other cancers [14,15]. In a recent Polish study, the prevalence of depression among lung cancer patients was 41.7% and of anxiety was 37.2% [16].

The topic of lung cancer among women was analyzed by us in some aspects previously. We discussed the specificity of lung cancer among women, including risk factors [6], and analyzed surgical treatment and survival in both sexes: women had a better long-term outcome compared to men, with no significant differences in disease severity [17]. We also focused on the characteristics of young women with lung cancer who were surgically treated [18] and we noticed that women under 55 years of age, despite surgical treatment in a more advanced stage of the disease, had less complications and a better 5-year survival compared to older women.

The aim of this study is an analysis of the clinical, sociodemographic and psychological aspects of female lung cancer. To better express the results, we compared women and men.

## 2. Methods

Consecutive patients admitted between January and March 2023 to the outpatient oncology clinic (Department of Lung Cancer and Chest Tumours, Maria Skłodowska-Curie National Research Institute of Oncology in Warsaw) and the Department of the Internal Medicine, Pulmonary Diseases and Allergy were enrolled. The following inclusion criteria (the same for women and men) were used: histopathological confirmation of lung cancer, history of lung cancer treatment, consent to participate and understanding the questions included in the questionnaires. The exclusion criterion was cognitive impairment indicative of dementia.

This study was approved by the Bioethics Committee at the Medical University of Warsaw.

Demographic data, smoking history, education, domicile, family history of cancer, primary symptoms, course of the disease and history or need for consultation with a psychologist/psychiatrist in terms of lung cancer were collected from a self-administered questionnaire. 

The patients were evaluated based on the eighth edition of the TNM classification for lung cancer [19], histological diagnosis according to the latest WHO recommendations [20], cancer mutation status and applied treatment according to medical records.

Psychological evaluation was obtained from self-administered questionnaires (the Perceived Stress Scale (PSS-10) and the Acceptance of Illness Scale (AIS).

The AIS was developed by Felton et al. [21] and adapted for Polish patients by Jurczyński [22]. The scale contains eight statements that describe the negative consequences of disease. The patients were required to determine their current condition using a five-grade scale (1—strongly agree and 5—strongly disagree). The sum of all points (ranges from 8 to 40) determines the overall assessment of the acceptance of the disease. Low scores (below 20 points) indicate a lack or poor acceptance of the disease, moderate scores (between 20 and 30 points) mean a moderate level of acceptance, while high scores (over 30 points) indicate complete acceptance of the disease [23].

The PSS-10 was created by Cohen [24] and adapted to Polish conditions also by Jurczyński [22]. It was designed to measure the degree to which situations in one’s life are rated stressful. The 10-item PSS asks respondents to rate how often they felt or thought in a particular way during the last month on a scale from 0 (never) to 4 (very often). The possible score ranges from 0 to 40 points (0–13 points indicate a low level of stress, 14–19 points a moderate level of stress and 20–40 points reflect a high level of perceived stress).

### Statistical Analysis

The analysis was performed using R 4.1.0 (R Foundation for Statistical Computing, Vienna, Austria) for environmental statistical computing. Data are presented as median followed by interquartile range or as number with percentage. For comparison between groups, Wilcoxon or Chi-square tests were used. The results of the tests were considered significant when the *p*-value was lower than 0.05. We also used logistic regression models to evaluate odds ratios associated with specific treatments in women compared to men among subjects with stage III or IV lung cancer.

## 3. Results

### 3.1. Study Group Description

A total of 100 patients with confirmed primary lung cancer (50 women and 50 men) were enrolled in this study. Both women and men had a similar age: 66 (59.5–71) vs. 65 (61–71.8). The vast majority of patients in both sexes were ever smokers (84% vs. 98%) with a median pack-years of 22.5 for females and 45 for males (*p* < 0.05). Despite being diagnosed with lung cancer, 14% of women and 24% of men continue to smoke. We found no statistically significant differences in the incidence of chronic obstructive lung disease (COPD), second-hand smoke exposure, education, residence or symptoms and the time from onset of symptoms to diagnosis.

Patients’ characteristics are presented in Table 1 and Table 2.

### 3.2. Comparison of Clinical Data Related to Lung Cancer

**Figure 1:**
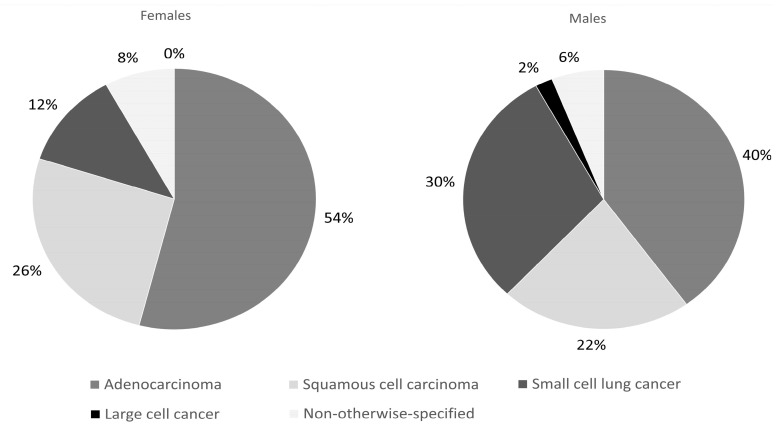
Histopathology diagnosis in both sexes.

**Figure 2:**
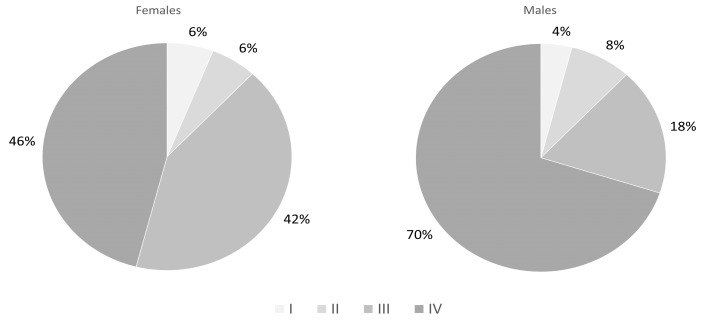
Stage of advancement of lung cancer in both sexes.

We found a statistically significant (*p* < 0.05) difference in the distribution of the histological type of lung cancer. However, in both sexes, the most common lung cancer was adenocarcinoma (54% vs. 40%), followed by squamous cell carcinoma (26% vs. 22%) and small cell lung cancer (12% vs. 30%). The occurrence of large cell cancer (8% vs. 2%) and non-otherwise-specified (NOS) cancer (0 vs. 6%) was low. The histopathology results of both sexes are shown in Figure 1. Activating mutations in the EGFR gene were found in nine patients (eight women and one man; *p* = 0.02) while rearrangements of ALK and ROS1 were reported in isolated cases. When evaluating the classification of TNM, we found a statistically significant difference in the distribution of clinical stage (*p* < 0.05), and the highest number of patients was classified as stage IV (46% vs. 70%)—detailed information is shown in Figure 2.

Within the history of treatment, women were significantly less likely to be treated with immunotherapy (*p* < 0.05), while in the other treatment methods, we found no statistically significant differences. Comparing treatment of subjects with stage III and stage IV lung cancer, we found that women had a lower probability of receiving immunotherapy as a cancer treatment (odds ratio 0.38 with 95% confidence interval 0.16–0.90; *p* = 0.03. Other methods of therapy are compared in Table 3—data are presented as crude odds ratios with 95% confidence intervals associated with female sex.

### 3.3. Psychological Assessment

The results of AIS were similar in both groups. In PSS-10, women and men obtained a score for moderate perceived stress; however, the women’s results were borderline for high stress (18.9). Furthermore, women expressed the need to seek psychological or psychiatrist consultation in terms of lung cancer diagnosis significantly more often (*p* < 0.05), but only a half of them finally did so. A history of the above-mentioned consultations was declared by 24% of women and 6% of men. The assessment of the mental state of women compared to men is presented in Table 4.

## 4. Discussion

We analyzed the clinical, sociodemographic and psychological aspects of female lung cancer. For a better characterization of our group, we compared it with a group of men. As a novelty, we took into account the psychological aspects of malignant disease. Our study confirmed well-known facts in relation to previous studies; however, the results of the comparisons between both sexes may be considered new. We found a significantly shorter history of smoking in the group of women; at the same time, there were no differences in the reported incidence of COPD between men and women. Significant differences in histopathological diagnosis were observed with the dominance of adenocarcinoma. The second most common type in women was squamous cell carcinoma, while in men it was small cell lung cancer. According to gene mutations, we only found differences in the presence of EGFR mutations, which were more frequent in women. The total frequency of EGFR mutations was similar to that previously reported in Caucasian NSCLC patients (women and non-smokers have a higher incidence) [25,26].

Despite comparable results to men on the psychological questionnaire (PSS-10, AIS), women more often reported a willingness to be supported by a psychologist or psychiatrist due to lung cancer. However, they did not decide to consult them more often than men. By comparing the treatment history, immunotherapy was found to be a significantly less frequently used method in women. However, in general, our study consolidates the knowledge that lung cancer is a serious problem among women.

The most important is the fact that most of the patients were in stage III or IV of lung cancer (in both sexes 88%). Unfortunately, the unfavorable distribution of advanced stages of lung cancer persists and the previous reports support our results [27]. Furthermore, we found a significant difference between women and men—more men than women had advanced lung cancer (stage IV). This is in contrast to global results—in general, the early stage of the disease (I–III) is more frequently diagnosed in men than in women (in both Europe and Asia) [28]. The type and advancement of lung cancer impact the methods of treatment. However, we did not observe differences related to sex when the rates of surgery, chemiotherapy, radiotherapy and targeted therapy were compared. The only difference found was the fact that immunotherapy was less frequent in women than in men. In terms of immunotherapy treatment, our study is consistent with the results of other authors. A lower rate of immunotherapy in women than in men was reported by Stabellini et al. [29]; they also noticed higher rates of surgery in women than in men, which is in contrast to our previous study where women were operated on less frequently compared to men [17]. In general, in the immunotherapy registration studies, the majority of patients were male [30,31,32].

According to the American Cancer Society, most patients are diagnosed with lung cancer at 65 years or older [33]. In the study conducted by Ruano-Ravina et al. [34], women were diagnosed on average 4 years earlier than men. We have not observed such differences. However, we found two women under 40 years of age in our group.

As we know, the clinical course of lung cancer is insidious and often asymptomatic for a long time. The most common reported symptom in women was cough (as in men). Ruano-Ravina et al. noted different observations in terms of symptoms—in their study, weight loss was the most common symptom in both sexes. However, we did not find differences in the reported symptoms between sexes and our results are consistent in this regard with the conclusion of Ruano-Ravina A et al., who showed a lack of differences between men and women in the symptoms of lung cancer at the time of diagnosis in general.

In relation to the risk factors that affect both the morbidity and course of lung cancer, we have found few differences. Women had a significantly shorter history of smoking than men with a higher percentage of non-smokers in the group (non-smoker women 16% vs. non-smoker men 2%) which is consistent with other studies [2,8]. In a multicenter case–control study, women had 20–25% lower average pack-years at diagnosis compared to men [35]—in our study, that difference was even higher. Overall, 14% of women and 24% of men continued to smoke despite the diagnosis of lung cancer. Daniel et al. reported similar results—17% of patients, mainly with advanced lung cancer, were defined as ‘persistent smokers’ [36] without sex division.

Second-hand smoke exposure is possible risk factor for lung cancer; however, the data concerning this factor are extremely scanty in clinical reports. Patients in both sexes reported exposure to second-hand smoke (68% women vs. 58% men). However, both groups had an important history of smoking (84% vs. 98%). The impact of passive smoking is also difficult to assess at the time of active exposure. Only four women were never smokers and had passive exposure to tobacco (in this subgroup, adenocarcinoma was diagnosed in three patients). Second-hand smoking is an important factor that increases lung cancer among non-smoking spouses by 20–30% [37]. 

In our study, eight women had a history of lung cancer (the same number was obtained for men) in first-degree relatives. In the group of women with a familial history, squamous cell carcinoma was diagnosed the most frequently (four women), then adenocarcinoma (two women), NOS and small cell lung cancer. The results of Kaoru Yoshida show that a family history of lung cancer in first-degree relatives was associated with an increased risk of lung cancer between both sexes. According to histology and the type of family members, a parental history of lung cancer was significantly associated with an increased risk of female adenocarcinoma [38]. 

COPD was the most common pulmonary comorbidity in subjects with lung cancer, and in our study it was similarly frequent in women (26%) and men (22%). In total, 45–63% of lung cancer patients globally are estimated to be affected by COPD [39]. Loganathan Raghu S and Wang W observed that the prevalence of COPD in women was significantly lower compared to men [40,41], which is in contradiction with our results. However, it is assumed that the number of COPD patients in Poland is underestimated. The results of many analyses show that COPD is diagnosed primarily in its severe and very severe forms [42]. Undrunas A studied the prevalence of COPD in the Polish lung cancer screening cohort and COPD was newly diagnosed in 20% of the patients (in the group of womenCOPD was diagnosed in 17%). Women with a diagnosis of COPD were younger than men and smoked fewer cigarettes [43].

Sociodemographics can have an impact on the survival and treatment of patients with lung cancer. People with a lower socioeconomic status have worse cancer survival, most likely due to the limited likelihood of receiving both traditional and next-generation treatments, and higher rates of comorbidities. They are generally diagnosed in the later stages and have a higher prevalence of smoking, but there is a chance that this may change due to the spread of lung cancer screening programs. However, early evidence suggests that there may occur socioeconomic inequalities in the use of these screening programs [12]. Data from studies involving large groups of patients have shown that low socioeconomic status remains a risk factor for lung cancer even after accounting for smoking habits. The strongest inverse relationship between lung cancer and socioeconomic status was observed in education [44,45]. Huaqiang Zhou et al. provided evidence to suggest that a higher education level plays a causal role in lowering the risk of lung cancer [46]. We observed a trend towards the prevalence of higher education levels among women with lung cancer, similar to Herndon JE [47].

Sociodemographic and clinical factors are also important for finding the cause of psychological distress among lung cancer patients. Xu Tian et al. presented a predictive algorithm based on sociodemographic and clinical factors to search for the risk of psychological distress among lung cancer patients. They included 19 factors, among which younger age, higher educational level, work, extremely low or high household income, shorter duration of diagnosis, lack of family history, drinking history and advanced cancer stage were first identified as risk factors for psychological distress [48]. The results of several studies showed that anxiety and depression in cancer patients occurred in 12–25%, with a higher prevalence reported in lung cancer, women and younger patients [49].

An important part of our project was the assessment of women’s mental state. We found that despite similar results to males on psychological questionnaires (PSS-10, AIS), women reported a willingness to be supported by a psychologist or psychiatrist due to lung cancer four times more often. However, ultimately, they do not decide to consult them more often than men. Female cancer patients in general are almost two times more likely than males to report clinical levels of depression. Women with lung cancer demonstrated the highest prevalence rate of depression, with 24.7%, compared to other cancers [50]. A series of recommendations have been distributed in cancer settings for routine active screening for distress among patients [51]. However, less than half of patients with advanced lung cancer with anxiety or depressive symptoms received mental health care [52,53].

AIS score was considered as an independent determinant of the physical and mental component of quality of life among lung cancer patients. Our results on the AIS scale show a moderate level of acceptance for lung cancer and are consistent with the study conducted by Chabowski [54], in which the mean score of acceptance of the disease was 27.1. Kaczmarek-Borowska et al. have published similar results, in which an average degree of acceptance was found in 56.16% of lung cancer patients [55]. Women obtained a higher median score in PPS-10 (18.9 vs. 16.3). The results of the patients of both sexes were within the range of medium stress; however, the results for women were borderline (high stress is defined as 19 or more points). In the study conducted by Tian X et al., the median score on this scale was 20 (71.4% of the group were men) [56]. Tian X et al. confirmed that perceived stress partially mediated the relationship between social support and psychological distress, with a positive correlation between perceived stress and psychological distress. Despite comparable results to men on psychological questionnaires (PSS-10, AIS), women reported a more frequent willingness to be supported by a psychologist or psychiatrist due to lung cancer. However, they do not decide to consult more often than men.

Despite some limitations (the relatively low number of patients, patients only in good condition qualified for treatment and data declared by patients in questionnaires), this study provided evidence for some important differences in sociodemographic, clinical and psychological characteristics associated with lung cancer among women.

## 5. Conclusions

A statistically significantly shorter history of smoking and a higher incidence of adenocarcinoma among women confirms the supposition of the importance of other factors with lung cancer. Future studies should investigate potential factors other than smoking among women in the field of lung cancer screening programs. Due to the lack of differences in the reported incidence of COPD between men and women, despite a clear contrast in the number of pack-years, the diagnosis of COPD should be considered more often among women. Physicians should be aware of the less frequent use of immunotherapy in women and improve access to this form of treatment. The problem of differences in lung cancer in women has been already noticed, which has been confirmed by the introduction of a special section dedicated to women in the SOLACE screening program (in Europe)—to increase women’s knowledge about lung cancer and their participation in such programs [57]. We should also be more active in finding out the willingness to consult a psychologist or psychiatrist among women.

## 6. Clinical Practice Points

1. The role of collecting sociodemographic information among lung cancer patients continues to grow.
2. Appropriate campaigns of screening programs aimed at different education levels in both sexes are needed.
3. We should be more active in finding out the willingness to consult a psychologist or psychiatrist among women with lung cancer.
4. The diagnosis of COPD should be considered more often among women due to the lack of differences in the reported incidence of COPD between men and women, despite a clear contrast in the number of pack-years.

## Figures and Tables

**Table 1:** The comparison of study participants.

	Females (N = 50)	Males (N = 50)	*p*-Value
Age (years)	66 (59.5–71)	65 (61–71.8)	0.939
Pack-years of smoking	22.5 (6.2–30)	45 (26.2–50)	<0.05
Current smokers (N, %)	7 (14%)	12 (24%)	<0.05
Ever smokers	42 (84%)	49 (98%)	
Non-smoker status (N, %)	8 (16%)	1 (2%)	
Second-hand smoke exposure (N, %)	34 (68%)	29 (58%)	0.407
COPD (N, %)	13 (26%)	11 (22%)	0.815
Primary symptoms			0.745
Cough (N, %)	32 (64%)	28 (56%)	
Dyspnoea (N, %)	16 (32%)	14 (28%)	
Weight loss (N, %)	11 (22%)	12 (24%)	
Chest pain (N, %)	8 (16%)	8 (16%)	
Hemoptysis (N, %)	3 (6%)	3 (6%)	
Hoarseness (N, %)	6 (12%)	11 (22%)	
Symptoms of pneumonia (N, %)	6 (12%)	5 (10%)	
Time from onset of symptoms to diagnosis (months)	5 (2–6)	3 (2–6)	0.416
Histopatology diagnosis			<0.05
Adenocarcinoma (N, %)	27 (54%)	20 (40%)	
Squamous cell carcinoma (N, %)	13 (26%)	11 (22%)	
Small cell lung cancer (N, %)	6 (12%)	15 (30%)	
Large cell cancer (N, %)	4 (8%)	1 (2%)	
Not otherwise specified	0 (0%)	3 (6%)	
EGFR mutations (N, %)	8 (29.6%)	1 (4.3%)	<0.05
ALK rearrangements (N, %)	2 (7.4%)	1 (4.3%)	0.649
ROS1 rearrangements (N, %)	1 (3.7%)	0 (0%)	0.351
Clinical stage			<0.05
I (N, %)	3 (6%)	2 (4%)	
II (N, %)	3 (6%)	4 (8%)	
III (N, %)	21 (42%)	9 (18%)	
IV (N, %)	23 (46%)	35 (70%)	
Treatment			
Surgery (N, %)	10 (20%)	7 (14%)	0.594
Chemiotherapy (N, %)	35 (70%)	40 (80%)	0.421
Radiotherapy (N, %)	20 (40%)	25 (50%)	0.356
Immunotherapy (N, %)	13 (26%)	25 (50%)	<0.05
Targeted therapy (N, %)	9 (18%)	4 (8%)	0.25

COPD—chronic obstructive pulmonary disease. Data presented as number of cases (% all cases) or median followed by interquartile range.

**Table 2:** Sociodemographic comparison of study participants.

	Females (N = 50)	Males (N = 50)	*p*-Value
Education level			0.053
Lower (N, %)	6 (12%)	8 (16%)	
Secondary (N, %)	21 (44%)	31 (62%)	
Higher (N, %)	22 (44%)	11 (22%)	
Residence			0.165
Village (N, %)	10 (20%)	13 (26%)	
City to 50,000 (N, %)	10 (20%)	10 (20%)	
City 50–100,000 (N, %)	2 (4%)	9 (18%)	
City 100–500,000 (N, %)	3 (6%)	3 (6%)	
City > 500,000 (N, %)	23 (46%)	15 (30%)	
Family history of lung cancer	8 (16%)	8 (16%)	1

**Table 3:** Odds ratio with 95% intervals for receiving specified methods of treatment associated with female sex in subjects with 3 or 4 stage lung cancer.

Treatment Method	Odds Ratio with 95% Confidence Interval	*p*-Value
Surgery	1.05 (0.27–4.05)	0.94
Chemotherapy	0.58 (0.21–1.54)	0.27
Radiotherapy	0.63 (0.27–1.45)	0.28
Immunotherapy	0.38 (0.16–0.90)	0.03
Targeted therapy	2.7 (0.81–10.6)	0.12

**Table 4:** Assessment of women’s mental state compared to men’s.

	Females (N = 50)	Males (N = 50)	*p*-Value
Need of consultation with psychologist/psychiatrist in terms of lung cancer	12 (24%)	3 (6%)	<0.05
History of using a psychologist’s/psychiatrist’s consultation in terms of lung cancer	6 (12%)	3 (6%)	0.485
PSS-10	18.9 (12.7–25.1)	16.3 (10.1–22.5)	<0.05
AIS	27 (22–32)	27 (20.2–31)	0.648

PSS-10—Perceived Stress Scale; AIS—Acceptance of Illness Scale. Data presented as number of cases (% all cases) or median followed by interquartile range.

## Data Availability

The datasets used and/or analyzed during the current study are available from the corresponding author on reasonable request.

## Abbreviations

AIS	Acceptance of Illness Scale
ALK	anaplastic lymphoma kinase
BRAF	v-raf murine sarcoma viral oncogene homolog B1
COPD	chronic obstructive pulmonary disease
EGFR	epidermal growth factor receptor
NOS	non-otherwise specified
PSS-10	Perceived Stress Scale
ROS1	ROS proto-oncogene 1, receptor tyrosine kinase
TNM	Tumor, Node, Metastasis
